# Severe acute malnutrition in children admitted in an Intensive Therapeutic and Feeding Centre of South Kivu, Eastern Democratic Republic of Congo: Why do our patients die?

**DOI:** 10.1371/journal.pone.0236022

**Published:** 2020-07-17

**Authors:** Richard Mbusa Kambale, Gaylord Amani Ngaboyeka, Joseph Ntagerwa Ntagazibwa, Marie-Hélène Igega Bisimwa, Lisa Yvette Kasole, Viateur Habiyambere, Victor Buunda Kubuya, Joseph Kasongowa Kasongo, Emmanuel André, Dimitri Van der Linden

**Affiliations:** 1 Institute of Experimental and Clinical Research, Université Catholique de Louvain, Brussels, Belgium; 2 Université Catholique de Bukavu, Bukavu, Democratic Republic of Congo; 3 Pediatric Department, Hôpital Provincial Général de Référence de Bukavu, Bukavu, Democratic Republic of Congo; 4 Laboratory of Clinical Bacteriology and Mycology, Department of Microbiology and Immunology, KU Leuven, Leuven, Belgium; 5 Pediatric Infectious Diseases, General Pediatrics, Pediatric Department, Cliniques Universitaires Saint Luc, Brussels, Belgium; Kemri Wellcome Trust, KENYA

## Abstract

**Background:**

Severe acute malnutrition (SAM) remains a serious public health concern in low- and middle-income countries. Little is known about treatment outcomes of child inpatients in Intensive Therapeutic and Feeding Units. This study aimed to assess treatment outcomes of SAM and identify factors associated with mortality among children treated at Saint Joseph Nutritional Center, South Kivu, Eastern Democratic Republic of Congo.

**Methods:**

A retrospective hospital-based cross-sectional study was conducted on medical records of 633 severely malnourished children followed as inpatients at Saint Joseph Nutritional Center from July 2017 to December 2018. Data were entered, thoroughly cleaned and analyzed in SPSS version 25. Univariable and multivariable logistic regression model were fitted to identify factors associated with mortality.

**Results:**

Among 633 patients admitted with SAM, 13.1% were lost to follow-up and 9.2% died while in hospital. Children with late referral to the health facility (> 14 days) after the onset of main external malnutrition signs had 2.03 times higher odds of death than those referred less than 14 days [AOR = 2.03 at 95%CI (1.12, 3.68)]. The odds of death was 1.91 times higher for children with MUAC < 115 mm than for those with MUAC ≥ 115 mm [AOR = 1.91 at 95% CI (1.05, 3.50)]. Children infected with HIV were 3.90 times more likely to die compared to their counterparts [AOR = 3.90 at 95% CI (2.80, 9.41)].

**Conclusion:**

Particular emphasis should be placed on partnering with communities to improve information on malnutrition signs and on critical importance of early referral to the health system. While HIV incidence in DRC is still low (0.21%), its impact on mortality among severely malnourished children is increased due to the limited access to HIV testing and antiretroviral therapy.

## Introduction

Tremendous progress has been made worldwide in child survival in recent decades. Among children under-5, nine percent of children died in 1990 compared to four percent in 2017. The under-five mortality rate fell from 93 deaths per 1,000 live births in 1990 to 39 in 2017 [1].

Despite all this progress, 5.4 million children under-5 still die each year, 80% of them in sub-Saharan Africa and South Asia. Almost half of these deaths occur among malnourished children [2–4]. Malnutrition is a serious public health concern in low- and middle-income countries, and is the focus of the first World Health Organization (WHO) Millennium Development Goal. In its acute form, it affects about 50 million children under-5 worldwide, including 48 million in Africa and Asia [4].

Acute malnutrition is subdivided into 2 categories: (i) Moderate Acute Malnutrition (MAM), defined as mid-upper arm circumference (MUAC) between 115 and 125 mm in children aged 6–59 months; or a weight-for-height/length z-score between –2 and –3 according to the 2006 WHO growth standards; with absence of edema; (ii) Severe Acute Malnutrition (SAM), defined by two distinct clinical entities: (a) non-edematous malnutrition (marasmus), defined as MUAC < 115 mm in children aged 6–59 months; or a weight-for-height/length z-score < –3 according to the 2006 WHO growth standards; (b) edematous malnutrition (kwashiorkor), defined by bilateral pitting edema [5].

SAM is further classified according to the presence or absence of medical complications. Uncomplicated SAM is defined as SAM with no apparent signs of infection nor other signs requiring hospitalization, and with adequate appetite. Complicated SAM is defined as SAM with clinical signs of infection, metabolic disorders, severe edema, hypothermia, vomiting, severe dehydration, profound anemia or lack of appetite [5].

SAM impairs life, cognitive and neurodevelopmental prognosis of children, especially in in low- and middle-income countries. The shorter-term prognosis of SAM depends on the type of malnutrition. In uncomplicated SAM, the mortality rate is < 5% while it varies from 10 to 40% in complicated SAM [6]. Further, previous reports have shown high post-discharge mortality following in-hospital management of diarrhea-SAM, HIV infection-SAM and pneumonia-SAM comorbidities respectively [7–9]. The FuSAM (Follow Up of Severely Acute Malnourished Children) study reported a mortality rate of 25% over a year after the SAM treatment among children followed in inpatient-based SAM treatment centre in Blantyre (Malawi). The risk of mortality after hospital discharge was fourfold higher for HIV-SAM compared with HIV-negative children with SAM [10]. In addition, ChroSAM (Chronic disease Outcomes after Severe Acute Malnutrition) study, which followed Malawian children with SAM, seven years post-discharge in the same centre, reported that children had poorer growth, body composition and physical function compared with siblings and community controls. These parameters are all risk factors of future cardiovascular and metabolic disease [11].

According to World Food Program (WFP), more than 6 million people are in a situation of acute food insecurity and livelihood crisis in the Democratic Republic of Congo (DRC) [12]. The combination of persistent violent armed conflicts, massive populations displacements, poor or inexistent infrastructures and widespread deterioration of productive assets have significantly affected food security in the DRC, and particularly in Eastern provinces, over the past two decades [12]. The impact of all these on children under-5 in the South Kivu province are alarming: the stunted rate of 48%, the wasting rate of 3% and the under-5 mortality rate of 4% [13].

Since 2000, joint efforts of the WHO and the United Nations Children’s Fund (UNICEF) allowed the standardization of the management of acute malnutrition which improved outcomes and prevented complications [14, 15]. Following these recommendations, the Ministry of Public Health of DRC has implemented the Integrated Management of Acute Malnutrition programme (Prise en Charge Intégrée de la Malnutrition Aigüe, PCIMA) [16]. PCIMA approach encompasses three components: (i) community outreach and mobilization; (ii) outpatient management of SAM without medical complications; and (iii) inpatient management of SAM with medical complications. Children with complicated SAM are managed in Intensive Therapeutic and Feeding Unit, which is one of the units of the pediatric department.

This study aimed to describe the treatment outcomes and to identify risk factors associated with mortality for a cohort of children admitted with SAM in a Nutritional Center located in the South Kivu province of DRC.

## Methods

### Study setting

This study was performed at Saint Joseph Nutritional Centre (SJNC), which is an Intensive Therapeutic and Feeding Unit included in the department of Pediatrics, of the provincial reference hospital (Hôpital Provincial Général de Réference de Bukavu, HPGRB). HPGRB hospital is the main healthcare facility in Bukavu, a city of more than 700,000 inhabitants and is the teaching hospital of the Université Catholique de Bukavu (U.C.B.).

The SJNC is specialized in the management of children severely acutely malnourished referred mainly from rural and urban settings surrounding Bukavu. It includes an intensive care unit (5 beds), a transition unit (15 beds), a nutritional rehabilitation unit (35 beds) and an isolation unit (5 beds). Children are admitted to the intensive care unit or transition unit according to the severity of medical complications, or to the nutritional rehabilitation unit for cases with mostly nutritional problems. Children with contagious diseases are admitted to the isolation unit. The staff is constituted by 3 medical doctors, including a pediatrician, 7 nurses and 1 nutritionist. The Centre admits an average of 50 to 60 cases of malnutrition monthly. The average length of stay is approximately 3 weeks.

The SJNC is normally intended to manage complicated SAM. Sometimes, MAM and uncomplicated SAM cases are referred to the SJNC for nutritional management because the health centers intended to manage those cases are confronted with stockouts in therapeutic supplies. In addition, in SJNC, children admitted for complicated SAM are often accompanied by other siblings, themselves with MAM or uncomplicated SAM. Thus, in the SJNC, the majority of cases are complicated SAM and some cases of uncomplicated SAM and MAM.

Within SJNC, therapeutic consumables are provided by the National Nutrition Programme, supported by UNICEF. However, the implementation of WHO recommendations is hampered by many frequent stockouts due to the weakness of the national distribution system. Locally, International Committee of the Red Cross and WFP provide therapeutic milk formula (F-75 milk and F-100 milk) and Ready-to-Use Therapeutic Foods (RUTF), but these interventions remain insufficient to cover the nutritional consumables needs. During all the study period, SJNC was without the standard supplies. During these long periods of shortage, health caregivers use whole milk / maize-sorghum-soya (MASOSO)-vegetal oil-sugar porridge preparations in SAM management. This preparation is constituted of whole milk (in initial stabilization phase), and MASOSO (60g), vegetable oil which is derived from palm oil (10g) and sugar (10g) (in nutritional rehabilitation phase). Eighty grams of MASOSO vegetable oil-sugar flour mixture provides 357.3 Kcal. Vitamin and mineral supplements are also given when whole milk is used during this stockouts period.

### Study design, period and population

We conducted a retrospective hospital-based cross-sectional study on the medical records of malnourished children admitted to SJNC from July 2017 to December 2018. During this period, 834 children from 1 month to 18 years of age were admitted to the Centre. Among these, 201 (24%) were excluded from this analysis because they presented a moderate form of malnutrition (MAM). In total, we included 633 severely malnourished children, among which 612 were classified as complicated and 21 uncomplicated.

### Inclusion and exclusion criteria and clinical management

The study included medical records of SAM children between 1 month and 18 years of age. SAM in infants under 6 months of age was defined as weight-for-length z-score < –3, or the presence of bilateral pitting edema. In children between 6–59 months of age, it was defined as weight-for-height/length z-score < –3, or MUAC <115 mm, or presence of bilateral edema. In children between 5–18 years of age, it was defined as weight-for-height < 70% of the median (National Center for Health Statistics reference) or presence of bilateral edema. Children under one month of age and those with other causes of edema, such as renal or cardiac conditions were excluded.

At SJNC, children are treated according to WHO therapeutic guidelines for complicated SAM [15] adapted by PCIMA 2016 [16]. Standard inpatient management of SAM involves two phases: (i) initial stabilization when life-threatening complications are treated; (ii) nutritional rehabilitation when catch-up growth occurs. Dietary treatment includes 8 daily meals of F-75 milk for stabilization in intensive care or transition unit, followed by F-100 milk or RUTF (plumpy nut) for nutritional rehabilitation in cases exiting intensive care prior to discharge. These formulations are specifically conceived for the dietary treatment of severe malnourished patients and contain 75 Kcal/100 ml, 100 Kcal/100 ml and 500 Kcal/92g, respectively for F-75 milk, F-100 milk and plumpy nut sachet. Amoxicillin, 50-100mg/kg in two doses per day per os is given systematically, or parenteral ceftriaxone 100mg/kg once daily in cases of suspected severe or complicated infectious syndrome. Treatment is modified based on indications such as non-improvement of clinical condition and/or results of bacterial culture and antibiotic sensitivity testing.

Children are discharged from the Intensive Therapeutic and Feeding Unit if they fulfilled the following criteria: adequate appetite and consumption of RUTF; rising weight curve; no edema; no signs of infection; not under active treatment with injectable antibiotics; mother able to attend regular weekly outpatient sessions at Outpatient Feeding and Therapeutic Unit.

### Data collection and variables definition

The data were collected using a survey form. The dependent variable was the treatment outcome, encompassing the recovery rate, loss to follow-up rate, average daily weight gain, median length of stay and mortality rate. These were compared to Sphere Standards: mortality rate > 75%, default rate < 15%, average daily weight gain > 10%, length of stay between 14–42 days, and lost to follow-up rate < 10%. The independent variables were socio-demographic and variables related with clinical condition at admission and follow-up.

Fever, hypothermia, tachypnea, bradypnea, tachycardia, bradycardia, hyperleukocytosis, severe anemia and hypoglycemia were defined in reference to pediatric standards [17, 18]. Diarrhea was defined as at least three watery stools in less than 24 hours, with or without blood [19]. Severe dehydration was considered as the presence of 2 or more of the following: lethargy, sunken eyes, drinks poorly or not able to drink and skin pinch goes back very slowly [20]. Malaria was defined as a thick blood film positive for malaria parasites, any density. Bacteremia was defined as a laboratory-confirmed bloodstream infection with a pathogen cultured from one or more blood cultures [21]. Culturing more than 10^5^ colonies/mL of a single organism in the urine culture (with clean catch midstream urine sample) was considered as Urinary Tract Infection (UTI) [22]. Infectious diarrhea was diarrhea due to an infectious etiology. Due to the unavailability of early virologic diagnostic tests, HIV infection was defined either on the basis of positive HIV serology after 18 months of age or on the basis of clinical signs suggestive of HIV infection before 18 months, according to the WHO classification in children born to HIV-positive mothers [23]. Blood count, thick blood film for malaria, urinalysis and/or urine culture, ova and parasite examination and/or stool culture were often performed at admission.

Three grades of edema severity were distinguished: edema + (mild edema), located to both feet/ankles; edema ++ (moderate edema), situated on feet and lower legs, hands or lower arms; and edema +++ (severe edema), generalized to feet, legs, hands, arms and face [15].

Nutritional recovery was considered when severely acute malnourished children achieved the target weight ≥ 85% of weight-for-height/length z-score at discharge. Additionally, edema for consecutive 10 days should not be observed.

The average daily weight gain and the median length of stay were calculated only for cured discharged children. The average daily weight gain (in g/kg/day) was calculated differently according to whether it was marasmus or kwashiorkor. In children with marasmus, it was calculated using the formula:

$$\frac{Weight\;(in\;grams)\;at\;discharge\;-\;Weight\;(in\;grams)\;at\;the\;beginning\;of\;the\;feeding\;process}{Weight\;(in\;kg)at\;the\;beginning\;of\;feeding\;process\;x\;length\;of\;stay\;(in\;days)}$$

[24–26]

In children with kwashiorkor, it was calculated by the formula:

$$\frac{Weight\;(in\;grams)at\;discharge\;-\;Weight\;(in\;grams)\;at\;the\;edema\;resolution}{Weight\;(in\;kg)at\;the\;edema\;resolution\;x\;length\;of\;stay\;(in\;days)}$$

[24–26]

The threshold used was the one proposed by WHO: poor (average daily weight gain < 5g/kg/d), moderate (average daily weight gain between 5-10g/kg/d) and good (average daily weight gain >10g/kg/d) [24–26].

The threshold used to assess the mortality rate was the one proposed by WHO: unacceptable (mortality rate > 20%), poor (mortality rate between 11–20%), moderate (mortality rate between 5–10%) and acceptable (mortality rate < 5%) [24–26].

Loss to follow-up was defined by child absence for more than 3 days while in treatment [24–26].

### Statistical analysis

The data were checked for completeness and inconsistencies, entered, coded, cleaned and analyzed using SPSS for Windows Version 25 (SPSS Inc. Version 25.0, Chicago, Illinois). Severely malnourished descriptive characteristics were summarized as mean and standard deviation for normal continuous variables, or as median and range for non-normal continuous variables, and as number or percentages for categorical variables. For categorical variables, we compared proportions using the chi-square or Fischer exact test; for continuous variables, medians were compared using the Wilcoxon rank-sum test.

To study the factors associated with mortality, we conducted univariable and multiple logistic regression analyzes. The variables were imported into the multiple regression model on the basis of a value p ≤ 0.25 and/or on the basis of biological plausibility. The unadjusted and adjusted odds ratios with their 95% confidence intervals were used to measure the association between the variables and mortality.

### Ethical considerations

Ethical approval was granted by the Ethics Committee of the Université Catholique de Bukavu, DRC. The Ethical Committee waived the requirement for informed consent. All data were fully anonymized before we accessed them.

## Results

We analyzed medical records of 633 severely malnourished children admitted to SJNC during study period. Their characteristics are summarized in Table 1. SAM was more prevalent among children aged 6 to 59 months (512/625; 81.9%) compared to those combined under 6 months and over 59 months (p<0.05). Three hundred and thirty-two (51.2%) children were male and the sex-ratio (M/F) was 1.1. More than half of the severely malnourished children (358/618; 58.0%) came from urban areas. Overall, edematous malnutrition was the most prevalent form of SAM, found in 408 (65.3%) children.

**Table 1:** Baseline characteristics of the study population.

Characteristics	n (%)	
**Age (months), median (range) (n = 625)**		23 (1–192)
< 6	28 (4.5)	
6–24	335 (53.6)	
25–59	177 (28.3)	
> 59	85 (13.6)	
**Gender (n = 627)**		
Male	332 (52.9)	
Female	295 (47.1)	
**Health area of provenance (n = 618)**		
Rural	260 (42.0)	
Urban	358 (58.0)	
**History of malnutrition (n = 604)**		
No	403 (66.7)	
Yes	201 (33.3)	
**Time between the onset of main external malnutrition signs and first visit to health facilities (days), median (range) (n = 594)**		36 (7–85)
**Child's immunization status (n = 550)**		
Child fully immunized	355 (64.5)	
Child not fully immunized	195 (35.5)	
**Type of SAM (n = 625)**		
Non-edematous malnutrition	217 (34.7)	
Edematous malnutrition	408 (65.3)	
Edema +++	73 (11.7	
Edema ++	255 (40.8)	
Edema +	80 (47.5)	
**Antimicrobial before admission to SJNC/HPGRB (n = 568)**		
None	148 (26.0)	
At home	175 (30.9)	
At the health centre	245 (43.1)	
**Clinical and biological signs at admission (n = 544)**		
Fever	64 (11.7)	
Hypothermia	18 (3.3)	
Tachypnea	222 (40.8)	
Bradypnea	8 (1.5)	
Tachycardia	203 (37.3)	
Bradycardia	31 (5.7)	
Hyperleukocytosis/neutrophilia	41 (7.5)	
Severe anemia	43 (7.9)	
Hypoglycemia	72 (13.2)	

HPGRB: Hôpital Provincial Général de Référence de Bukavu; MUAC: mid-upper arm circumference measure; SAM: severe acute malnutrition; SD: standard deviation; SJNC: Saint Joseph Nutritional Center.

Four hundred (63.2%) children were brought to health facility for consultation more than 2 weeks after the onset of main external malnutrition signs (thinning, edema or yellow-coloured hair). The median time between the onset of main external malnutrition signs and the referral to health facilities was 36 days (range: 7–85). It was more important for children coming from rural areas (42 days) than those coming from urban ones (22 days) (p<0.05).

The anthropometric parameters of children late referred to the health facility (more than one month) were severely deteriorated (MUAC < 110 mm for non-edematous malnutrition and edema +++ for edematous malnutrition), compared to those of children early referred to the health facility (p<0.05).

**Fig 1:**
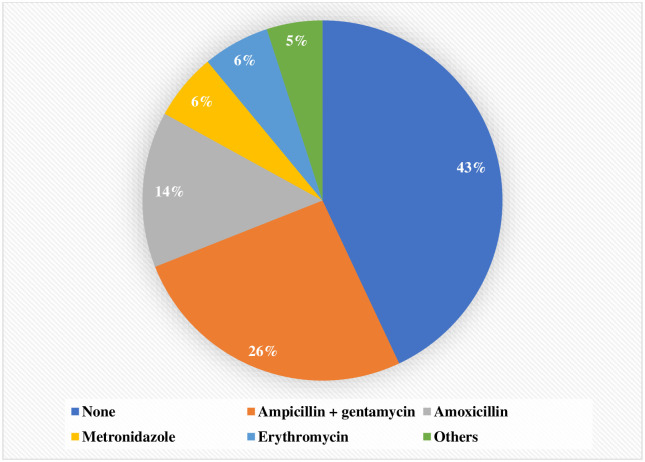
Antimicrobials used in severely malnourished children before their admission to the SJNC/HPGRB hospital (n = 568).

Four hundred and twenty (74.0%) children had received antimicrobials before admission to SJNC, including 175 (30.9%) at home. As shown in Fig 1, ampicillin and gentamycin were the most commonly administered antimicrobials at health centre (26%), while amoxicillin was the most commonly administered antimicrobial at home (14%). No child with uncomplicated SAM received antimicrobials before admission to SJNC.

**Fig 2:**
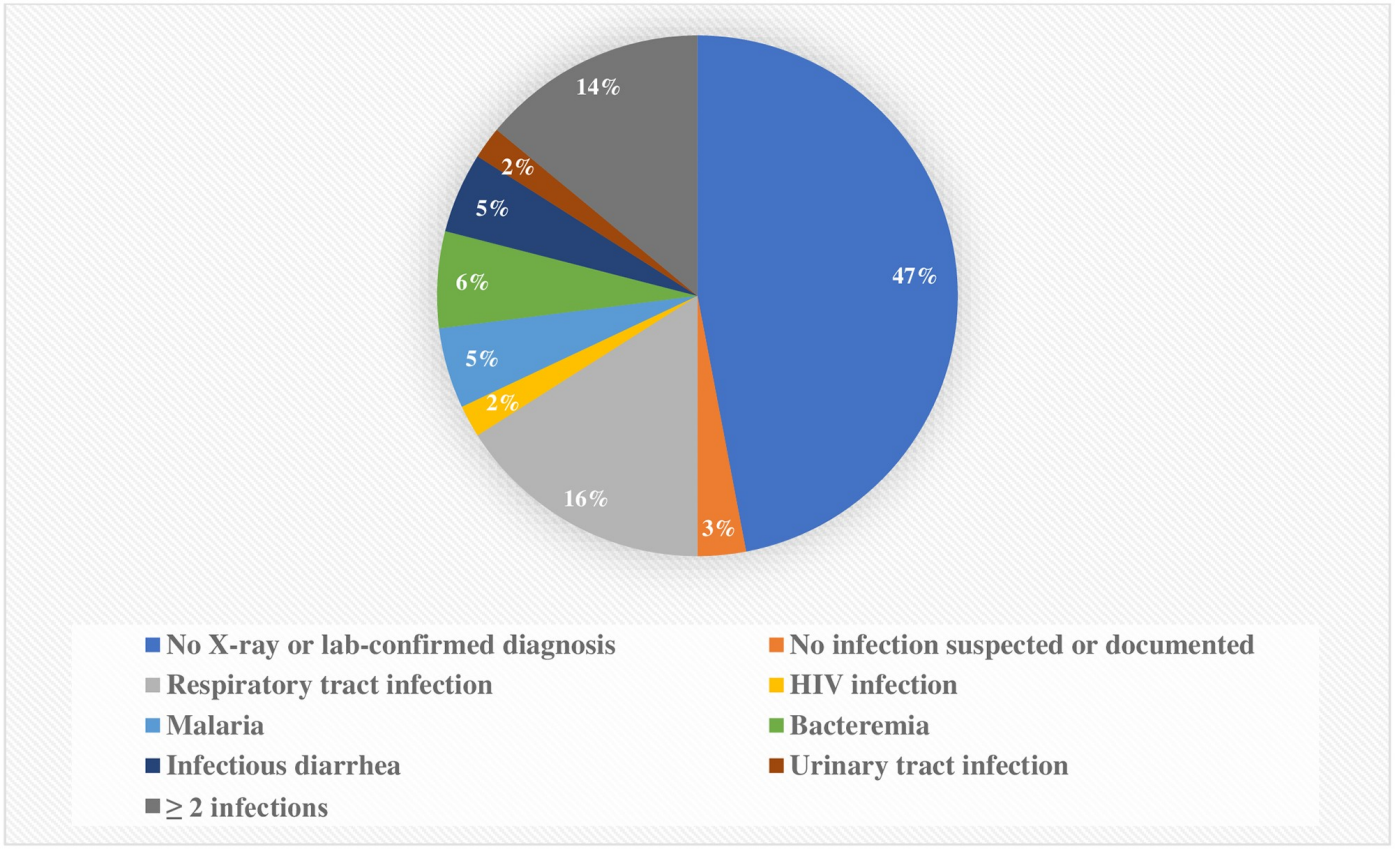
Main infectious diagnoses of severely malnourished children at SJNC/HPGRB hospital, June 2017–December 2018 (N = 633).

Fig 2 shows the distribution of main infectious diagnoses of severely malnourished children at SJNC. Overall, respiratory tract infections (16%), bacteremia (6%), infectious diarrhea (5%) and malaria (5%) accounted for 32% of confirmed diagnosis.

The average daily weight gain was 9.2 g/kg/day, higher in children with marasmus (10.3g/kg/day) than in children with kwashiorkor (8.2g/kg/day) (p<0.05). The median length of stay was 17 days (range: 8–28), longer in children with kwashiorkor (20 days) than in those with marasmus (11 days) (p < 0.05). Fifty-eight (9.2%) children died. As shown in Table 2, SJNC outcomes variables were comparable with that of international sphere standard (p> 0.05).

**Table 2:** Performance indicators of SJNC/HPGRB and sphere standard references.

Performance indicator	Indicator of SJNC/HPGRB hospital	International sphere standards reference	
		Acceptable	Alarming
Recovery rate, n (%)	492 (77.7) *	> 75%	< 50%
Mortality rate, n (%)	58 (9.2)	< 10%	> 20%
Default rate, n (%)	83 (13.1) *	<15%	> 25%
Average daily weight gain (SD) (g/kg/d)	9.2 (2.1) *	> 10%	< 5%
Length of stay (days), median (range)	17 (8–28) *	14–42	> 42
Lost to follow-up rate, n (%)	58 (9.2) *	< 10%	> 20%

*p > 0.05;

HPGRB: Hôpital Provincial Général de Référence de Bukavu (The Provincial Reference Hospital); SD: Standard Deviation; SJCN: Saint Joseph Nutritional Centre.

Variables associated with mortality at multivariable logistic regression included time between the onset of main external malnutrition signs and first consultation, MUAC and HIV infection. Severely malnourished children with delayed presentation to the health facilities (> 14 days) after the onset of main external malnutrition signs were 2.03 times higher odds of death than those referred less than 14 days [AOR = 2.03 at 95%CI (1.12, 3.68)]. The odds of death was 1.91 times higher for children with MUAC < 115 mm than for those with MUAC ≥ 115 mm [AOR = 1.91 at 95% CI (1.05, 3.50)]. Severely malnourished children infected with HIV were 3.90 times more likely to die compared to their counterparts [AOR = 3.90 at 95% CI (2.80, 9.41)] (Table 3).

**Table 3:** Bivariate and multivariable analysis of factors associated with mortality of severely malnourished children at SJNC/HPGRB hospital, July 2017–December 2018.

Variables and categories	Treatment outcome		OR (95%CI)	AOR (%CI)
	Dead n (%)	Cured n (%)		
**Health area of provenance**				
Rural	19 (11.2)	150 (88.8)	1.19 (0.66–1.88)	
Urban	38 (10.1)	340 (89.9)	1	
**Time between the onset of main external malnutrition signs and first visit to health facility (days)**				
< 7	7 (7.9)	82 (92.1)	0.36 (0.14, 0.93) **	
7–14	8 (21.6)	29 (78.4)	1	
> 14	25 (6.3)	375 (93.7)	3.46 (1.68, 7.12) ***	2.03 (1.12, 3.68) *
**Temperature**				
Hypothermia	6 (30.0)	14 (70.0)	4.5 (2.19, 9.56) ***	
Fever	4 (6.5)	58 (93.5)	0.97 (0.35, 2.65)	
Normal	30 (6.7)	420 (93.3)	1	
**Breathing rate**				
Bradypnea	4 (50.0)	4 (50.0)	6.5 (2.93, 14.4) ***	
Tachypnea	13 (6.1)	201 (93.9)	0.79 (0.41, 1.52)	
Normal	23 (7.7)	276 (92.3)	1	
**Type of SAM**				
Edematous malnutrition	7 (2.2)	308 (97.8)	0.14 (0.06, 0.31) ***	
Non-edematous malnutrition	34 (15.7)	183 (84.3)	1	
**MUAC**				
< 115 mm	27 (9.7)	250 (90.3)	2.21 (1.09, 4.47) **	1.91 (1.05, 3.50) *
≥ 115 mm	10 (4.4)	217 (95.6)	1	1
**Severe dehydration**				
Yes	14 (10.9)	115 (89.1)	1.58 (0.85–2.92) *	
No	27 (6.9)	367 (93.1)	1	
**Hyperleukocytosis/Neutrophilia**				
Yes	6 (14.6)	35 (85.4)	4.76 (1.03, 22.45) **	
No	2 (3.1)	63 (96.9)	1	
**HIV infection**				
Yes	5 (35.7)	9 (64.3)	5.28 (2.44, 11.46) **	3.9 (2.80, 9.41) *
No	34 (6.8)	464 (93.2)	1	1
**Bacteremia**				
Yes	5 (13.2)	33 (86.8)	1.81 (0.75–4.34) *	
No	36 (7.3)	459 (92.7)	1	
**Respiratory tract infections**				
Yes	8 (7.8)	94 (92.2)	1.02 (0.49–2.15)	
No	33 (7.7)	398 (92.3)	1	
**Diarrhea with dehydration**				
Yes	23 (9.9)	209 (90.1)	1.63 (0.89–2.94) *	
No	18 (6.1)	227 (93.9)	1	
**Malaria**				
Yes	30 (90.9)	3 (9.1)	0.98 (0.88–1.09)	
No	447 (92.5)	36 (7.5)	1	

*p < 0.25;

**p < 0.05;

***p < 0.01;

AOR: adjusted odd-ratio; HIV: human immunodeficiency virus; MUAC: middle upper arm circumference; OR: odd-ratio; SAM: Severe acute malnutrition; SJCN: Saint Joseph Nutritional Centre

## Discussion

The findings of this study provide preliminary data on factors associated with the mortality of severely malnourished children admitted as inpatients in the context of a food security crisis. Despite the unavailability of therapeutic supplies (RUTF, F-75 and F-100) at SJNC and their substitution by whole milk / MASOSO-vegetal oil-sugar preparations, the average daily weight gain of our study population was comparable with that of international sphere standard.

Several recent studies showed the non-inferiority of cereal-based formulas (maize, sorghum, soya) mixed with vegetable oil and sugar, in comparison to WHO conventional preparation, in the diet of severely malnourished children. A study conducted in North Kivu (Eastern DRC) comparing the efficacy and safety of two recipes (cow milk with porridge made of maize, soybean, vegetal oil and sugar versus WHO conventional preparation) among two groups of severely malnourished children, reported no significant difference between the two groups in terms of weight changes, edema resolution, gastrointestinal tolerability and clinical outcome over 21 days (p > 0.05) [27].

A non-inferiority, randomized controlled clinical trial was conducted in a rural area of South Kivu (Eastern DRC) to compare the efficacy of soya-maize-sorghum RUTF with that of standard peanut paste–based RUTF for treating SAM. Intention-to-treat and per-protocol analyses showed non-inferiority of soya-maize-sorghum RUTF compared with peanut paste–based RUTF for the recovery rate, weight gain and length of stay in children < 24 months of age [28].

Another non-inferiority individually randomized controlled efficacy clinical trial was conducted in Malawi to compare the efficacy of milk-free soya, maize, and sorghum enriched with crystalline amino acids to the standard peanut and milk RUTF. Intention-to-treat and per-protocol analyses showed non-inferiority for recovery rates and length of stay in children aged 24–59 months and 6–23 months between these two RUTFs [29].

The nutrient density of peanut and milk RUTF equivalent to WHO F-100 milk [14] ensures high rates of recovery and weight gain and low fatality rates [30, 31]. Nevertheless, peanut and milk RUTF high milk content (25%) makes it very expensive for sustainable use in low-and middle-income countries. About 50% of peanut and milk RUTF is still produced in high-income countries and imported into low-and middle-income countries where it is needed. The high cost of current standard RUTF (47$ USD for each child treated) remains, therefore, the main reason it is unavailable in resource-poor settings [32, 33]. The recipes based on soya, maize, and sorghum, vegetable oil and sugar has shown great promise and has the potential to provide a substantially lower-cost alternative, with adequate nutritional recovery.

The death rate found in our study was close to what is observed in similar settings. It remains, however, high compared to the PCIMA standards. Indeed, in non-emergency situations, PCIMA defines a mortality rate < 5% as an acceptable threshold [16]. This high death rate could be due to several barriers, often described in rural areas of low-and middle-income countries, including delayed referral to health facilities, mothers' lack of awareness of the signs of malnutrition, self-medication, or difficulties in accessing the health centre due to lack of transport. WHO often attributes infant or maternal mortality in low-and middle-income countries to the phenomenon known as "three delays": (i) delay in deciding to seek care, (ii) delay in identifying and reaching health facility, and (iii) delay in receiving adequate and appropriate treatment [34].

As in this study, MUAC as predictor of mortality in children with SAM has been described elsewhere [35–39]. Several studies [40–44] showed that MUAC may be used in preference over weight-for-height z-score for predicting the risk of death in under-5 children. In addition, these studies documented that use of MUAC < 115 mm alone may be better than weight-for-height z-score < -3 in identifying children at high risk of death. Recent evidence based on meta-analysis highlights that children with a weight-for-height z-score < -3 have about the same or higher risk of death as children with a MUAC < 115 mm. They both are at substantial risk of death, and neither should be omitted from protocols aimed at diagnosis and treatment of all SAM cases [45].

Our data showed a negative influence of HIV-malnutrition comorbidity on mortality. Delayed diagnosis and antiretroviral treatment (ART) initiation are factors that worsen these children's life-threatening prognosis. Indeed, for most mothers and children admitted to SJNC, HIV status is often unknown before admission. Secondly, almost all health facilities in Eastern DRC do not yet have early virologic diagnostic tests for HIV infection. HIV infection diagnosis is established either on the basis of positive HIV serology after 18 months of age or on the basis of clinical signs suggesting HIV infection before 18 months of age, according to WHO classification for children born to HIV-positive mothers. Finally, when HIV infection is diagnosed, ART is started only after these children have been stabilized nutritionally, according to the guidelines of the national AIDS care programme in DRC.

Recent WHO guidelines recommend early ART for all children, regardless of their clinical and immune status [46]. The rationale behind early ART for all children is that (i) early ART decreases significantly the mortality rate and improves better growth response than deferred ART (waiting for the CD4 count falls below 15%) [47]; (ii) early ART mitigates the negative effects of HIV infection on growth and pubertal and nervous system development [48–54] and (iii) it promotes immune recovery [55].

About 50% of admission diagnoses were not confirmed by radiography or laboratory tests. This is a major handicap in the framework of epidemiological surveillance. Where available, laboratory confirmation of clinical diagnoses is important to improve the accuracy of surveillance systems. In addition, although tuberculosis (TB) is common in immunodeficiency situations, including malnutrition, no children had a confirmed diagnosis of TB. In SJNC, treatment for TB is guided by a presumptive diagnosis based on the Keith-Edward score [56]. The factors contributing to uncertainties concerning incidence of tuberculosis in severely malnourished young children is related to the challenge in confirming a diagnosis: (i) difficulties in obtaining high quality specimens; (ii) children commonly have a poor bacillary count and many are negative on culture; (iii) the lack of mycobacterial culture facilities in settings where tuberculosis and malnutrition are endemic [57]. The Xpert MTB/RIF assay, a recent, rapid diagnostic test for the detection of M. tuberculosis and rifampicin resistance, has demonstrated encouraging results in the diagnosis of pulmonary tuberculosis in children [58]. Unfortunately, it is the appanage of only a few countries in low-income countries.

### Study limitations

The main limitation of study is its retrospective nature. Some medical records had incomplete data, increasing potential for selection bias.

## Conclusion

Our study reflects the current nutritional situation of the South-Kivu province in DRC, with a high burden of children affected by malnutrition. Particular emphasis should be placed on partnering with communities to improve recognition of the signs of malnutrition and on the critical importance of early entry into the health system. While HIV incidence in DRC is still low (0.21%) [59], its impact on mortality among severely malnourished children is increased due to the limited access to HIV testing and antiretroviral therapy.

## Supporting information

S1 Data (XLSX)

S2 Data (DOCX)
