# Supplementary material for: Severe acute malnutrition in children admitted in an Intensive Therapeutic and Feeding Centre of South Kivu, Eastern Democratic Republic of Congo: Why do our patients die?
Source: PLoS One. 2020 Jul 17;15(7):e0236022. doi: 10.1371/journal.pone.0236022 (PMC7367457; doi:10.1371/journal.pone.0236022)
Supplement: S2 Data — (DOCX) [file pone.0236022.s002.docx]

**CAPTIONS OF DATA**

1. Gender: 1= Male; 2 = Female
2. Health zone: 1= Kadutu; 2 = Bagira; 3= Ibanda; 4 = Rural zone; 1, 2 and 3 are urban health zones;
3. Socioeconomic level: 1= Low; 2= medium; 3= High; 4= Not reported;
4. Mother’s schooling level: 1= Illiterate; 2=primary; 3=Secondary; 4= University; 5= Manutention’s work; 6= Not reported
5. History of malnutrition: 1= Yes; 0=No
6. Child’s immunization: 1= Yes; 0= No
7. Antimicrobial before admission: 1=Home; 2= Health Center; 4=None
8. Type of antimicrobial: 1= Ampicillin + gentamycin; 2=Amoxicillin; 3=Metronidazole; 4=Erythromycin;
9. GB: White blood cell count
10. Diarrhea: 1=Yes; 0=No
11. Vomiting: 1=Yes; 0=No
12. Dehydration: 1=Yes; 0=No
13. Pneumonia: 1=Yes; 2=No
14. HIV: 1=Yes; 2=No
15. Malaria: 1=Yes; 2=No
16. Bacteremia: 1=Yes; 2=No
17. UTI (Urinary Tract Infection): 1=Yes; 2=No
18. Edema: 1= Yes; 2=No
19. Degree of edema: 1: Edema +; 2= Edema ++; 3= Edema +++
20. Discharge type: 1= Deceased; 2= Recovery; 3: Default
